# The effects of body hydration on perceptual responses during blood flow restriction exercise

**DOI:** 10.14814/phy2.70343

**Published:** 2025-04-22

**Authors:** Jenna C. McCrone, Christopher Pignanelli, Gavin C. Lydiate, Monica M. Grigore, Katrin Turetskiy, Alexa A. Robertson, Regan E. J. Parris, Ella M. Bisset‐Cavallin, Jamie F. Burr

**Affiliations:** ^1^ Department of Human Health and Nutritional Sciences University of Guelph Guelph Ontario Canada

**Keywords:** dehydration, discomfort, hypohydration, ischemia, metabolites

## Abstract

Blood flow restriction (BFR) training has emerged as a novel modality with clinical potential. However, BFR increases perceived effort and pain, highlighting the need to identify factors influencing perceptual responses to optimize its practical application. Hypohydration similarly increases discomfort during exercise or painful stimuli, but whether this interacts with BFR is unknown. The purpose of the study was to determine if hydration affects the perceptual response to BFR exercise. Of the 34 participants recruited, 17 (7 females) completed two BFR exercise bouts: (1) Hydrated (regular fluid intake) and (2) Hypohydrated (24 h fluid restriction). Rating of perceived exertion (RPE) and leg pain were recorded throughout. With hypohydration, urine specific gravity increased (Hydrated = 1.01 ± 0.009, vs. Hypohydrated = 1.025 ± 0.002, *p* < 0.0001), body mass decreased (−2.3 ± 0.7%, *p*  < 0.0001), and plasma volume decreased (−7.0 ± 3.4%, *p*  < 0.0001). Similar RPE and leg pain were reported during BFR exercise (RPE: 10.6 ± 0.9, vs. 11.1 ± 0.9, *p* = 0.054, leg pain: 3.5 ± 1.1, vs. 3.8 ± 1.2, *p* = 0.2). Similarly, during the rest periods, there was a minimal effect for RPE (9.1 ± 1, vs. 9.5 ± 1.3, *p* = 0.1) and leg pain (3.1 ± 1.5, vs. 3.6 ± 1.8, *p* = 0.09). Preliminary analyses show minimal sex differences in perceptual responses, with hydration status changes unrelated to BFR perception. Thus, hydration status has little impact on perceptual responses to BFR exercise.

## 1 INTRODUCTION

Blood flow restriction (BFR) exercise, which involves external limb compression to intentionally reduce flow, enhances strength and endurance adaptations at low resistance or intensity (Patterson et al., 2019). Notably, the external pressure applied during BFR does not need to completely cease blood flow into and out of the limb in order to facilitate training adaptations (Patterson et al., 2019). As such, BFR exercise offers potential as an alternative rehabilitation tool in situations where high external load or intensity exercise is restricted (Cognetti et al., 2022). However, a drawback of BFR exercise is the heightened sense of effort and perception of pain reported by participants, with recent evidence indicating that BFR accelerates these sensations in females more rapidly than in males (McClean et al., 2023). Given that perceived effort and pain can impact adherence to rehabilitation or training programs (Fisher et al., 1988; Jack et al., 2010), it is important to develop strategies and understand the factors influencing discomfort during BFR exercise to enhance its application in clinical settings.

The increased effort and pain during BFR exercise compared to traditional exercise is likely driven by the accumulation of metabolites (e.g., hydrogen ions, adenosine triphosphate, and lactate) in the restricted limb, which activate type III and IV nociceptive afferent nerves. In line with this reasoning, intramuscular injections of metabolites that simulate ischemic exercise have been reported to induce greater sensations of pain, though not fatigue, compared to free‐flow exercise (Pollak et al., 2014). While the role of type III and IV afferent nerves in pain perception is well‐established, their involvement in perceived effort remains debated. Meta‐analytic data from studies that partially blocked type III and IV afferent nerve feedback during exercise is inconclusive, suggesting that pain and effort should be studied separately when addressing discomfort in BFR exercise (Bergevin et al., 2023).

Experimental approaches have explored methods to reduce discomfort during BFR exercise, focusing on factors like tourniquet width (Rossow et al., 2012), pressure (Cerqueira et al., 2021; Weatherholt et al., 2013), and application cycle (Fitschen et al., 2014) have been shown to influence pain and/or effort perception. A common factor in these approaches is the manipulation of metabolite accumulation in the restricted limbs. Therefore, identifying modifiable variables that affect metabolite accumulation is crucial to minimizing unnecessary discomfort during BFR. Beyond tourniquet‐related factors, hydration status may also influence perceptual responses during BFR exercise. In fact, when hypohydrated, the rating of perceived exertion (RPE) increases during exercise (Logan‐Sprenger et al., 2015; Montain & Coyle, 1992) and the perception of pain increases to stimuli including cold temperature and post‐exercise ischemia (Bear et al., 2016; Tan et al., 2022). Therefore, the primary purpose of this study was to examine the effect of hydration status on perceived effort and pain during BFR exercise. We hypothesized that a state of hypohydration would increase both perceived effort and pain.

## 2 METHODS

### 2.1 Study overview

Thirty‐four young, healthy adults (males = 19, females = 15) were recruited to participate in the study, with only those achieving clear hypohydration included for analysis (see below). All potential participants completed a physical activity readiness questionnaire (PAR‐Q+), and provided written informed consent prior to commencing the study in accordance with approval from the University of Guelph research ethics board. The study involved three visits completed within 10 days: an introductory session to assess cardiorespiratory fitness and familiarize participants with BFR exercise, followed by two intervention visits which had participants arrive to the lab either hydrated or hypohydrated. Prior to each visit, participants abstained from alcohol/recreational drugs (48 h), intense exercise (48 h), low‐moderate intensity exercise (24 h), caffeine (12 h), supplements or nonessential over‐the‐counter medications (12 h), or food (2 h). Naturally menstruating females (*n* = 4) began the study in the early follicular phase of the menstrual cycle. Users of contraceptive pills (*n* = 7), patches (*n* = 0), or rings (*n* = 0) participated 1 week following menses. Participants with intrauterine device contraception methods that prevent typical bleeding patterns enrolled at any point of their cycle (*n* = 4).

### 2.2 Introductory visit

Height, body mass, and body composition were measured using bioelectrical impedance (Tanita InnerScan Body Composition Monitor, Tanita Corporation, Japan). Cardiorespiratory fitness was assessed using a graded exercise test on a cycle ergometer (Velotron, RaceMate, USA) with breath‐by‐breath indirect calorimetry (Quark CPET, COSMED, Italy). Starting at 20 watts, participants cycled at a self‐selected cadence with the load being increased by 20 watts steps each minute until exhaustion or a clear plateau in oxygen uptake despite increasing the power output. Maximal oxygen uptake (V̇O_2_ max) is reported as the highest 30 second rolling time average from the stage or verification phase. Participants were then familiarized with the BFR exercise as well as subjective rating scales (i.e., RPE and leg pain), as described below.

### 2.3 Intervention visits

**FIGURE 1:**
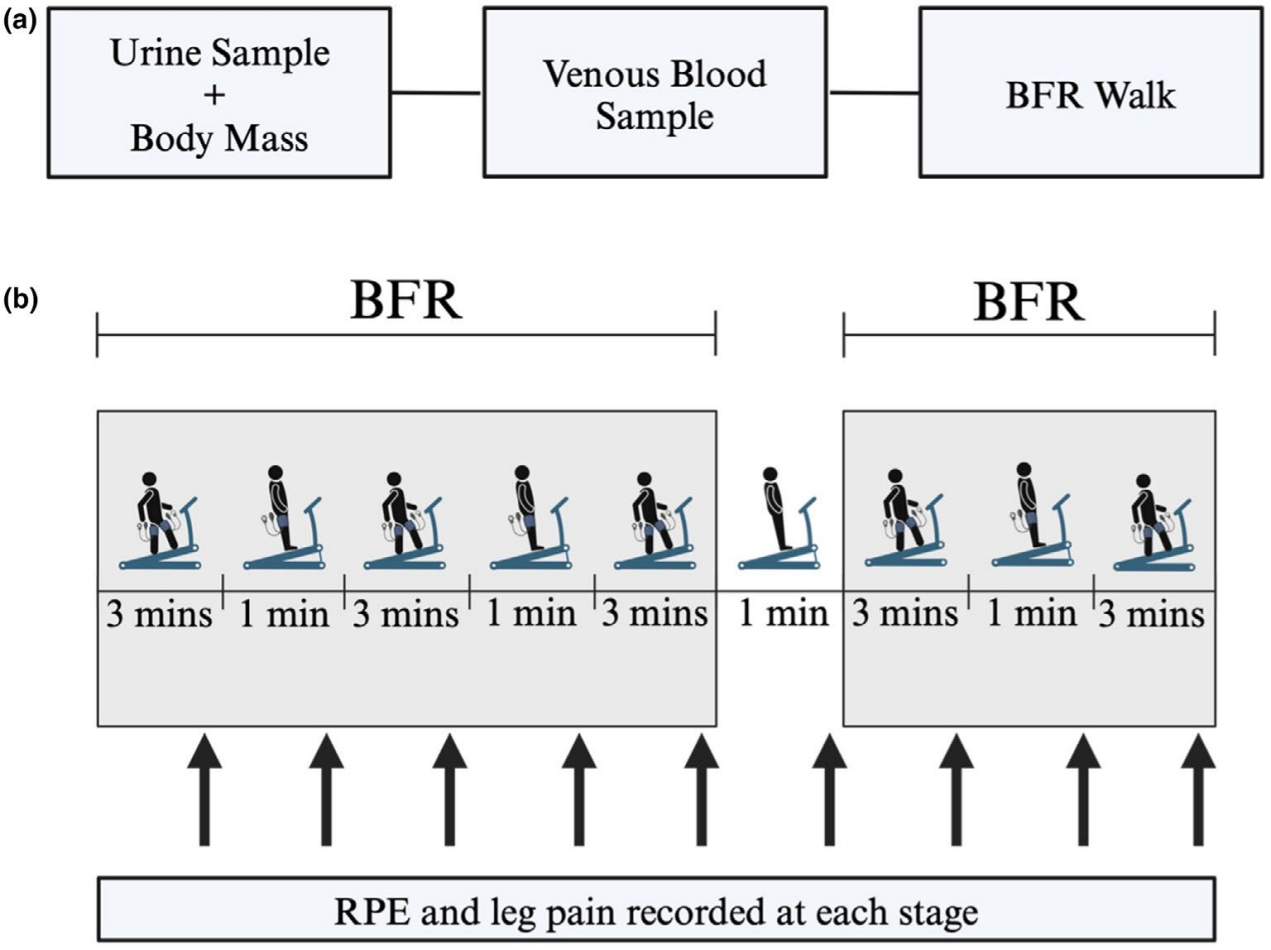
(a) Study overview. Urine, body mass, and venous blood samples were obtained prior to the blood flow restriction (BFR) exercise intervention. (b) Summary of the BFR walk protocol that included five 3‐min walk intervals at 5 km/h at a 5% incline interspersed with 1‐min of standing rest periods. Gray boxes indicate when BFR was applied during the protocol. Leg pain was determined using the Cook (0–11) scale and the rating of perceived exertion (RPE) was determined using the Borg (6–20) scale. Leg pain and RPE were taken at the mid‐point of each timepoint.

Participants completed two study visits, separated by a minimum of 48 h, either in a hydrated or hypohydrated state (Figure 1). Participants were encouraged to consume fluids throughout the day prior to the hydrated visit, or instructed to refrain from fluid consumption at least 24 h prior for the hypohydrated visit. Previous work supports a 24 h fluid restriction protocol that is sufficient to cause body hypohydration (Shirreffs et al., 2004; Tan et al., 2022; Tankersley et al., 1985). Refraining from fluid consumption provides a more ecologically valid method of inducing hypohydration, in contrast to more severe methods like diuretics (Armstrong et al., 1985). Upon arrival to the lab, a mid‐stream urine sample was provided for urine specific gravity (Atago Digital Hand‐Held Refractometer. Atago CO., LTD, Japan) and body mass was obtained. A venous blood sample was obtained from an antecubital vein after 30 min of seated rest for hematological analysis prior to performing the BFR exercise protocol. Temperature and relative humidity were kept similar between visits (Hydrated: 23.8 ± 1.0°C, 32 ± 9%; Hypohydrated: 23.6 ± 1.0°C, 33 ± 13%).

### 2.4 BFR exercise protocol

The BFR exercise protocol used in this study was selected based on prior research from our laboratory demonstrating its effectiveness for increasing V̇O_2_ max (Thompson et al., 2024). Limb occlusion pressure (LOP), synonymous with arterial occlusion pressure, is defined as the lowest pressure needed to cease blood flow into the limb at rest. The LOP was determined using an automated system (Personalized Tourniquet System, Delfi Inc. Canada) as an average of three measurements performed in a seated position using an 11 cm tourniquet. This oscillatory method of determining the LOP is similar to the gold‐standard method using Doppler ultrasound (Masri et al., 2016). Participants performed five 3‐min walks with BFR, separated by four 1‐min rest periods. The tourniquets were inflated throughout, except for a deflation during the 1 min of rest after the third walk bout. The treadmill speed was set at 5 km/h walking pace with a 5% incline.

The RPE and leg pain were collected at the mid‐point of each exercise and rest period. The RPE was obtained using the 6–20 Borg scale (Borg, 1982), and leg pain was quantified using the Cook‐11‐point scale (Cook et al., 1998). Participants were asked to read standardized instructions provided with the Cook‐11‐point. The pain scale uses numbers 0–10 and is designed to be used on only subjective feelings of pain (Cook et al., 1998). An additional 11th number (•) was provided on the scale which could be used by participants to rank their pain by any number larger than 10 (Cook et al., 1998). These instructions were used to minimize the possible crossover of different subjective perceptions (e.g., leg pain vs. RPE). Participants practiced using these scales during the introductory visit.

### 2.5 Hematological analysis

Hematocrit was measured in triplicate via microcapillary tubes (IEC Mirco‐Capillary Reader 2201, Damon Corporation, United States of America). The values were corrected for the blood sampling location (e.g., peripheral venous sampling) and plasma trapped between red blood cells (Chaplin et al., 1953; Chaplin & Mollison, 1952). Hemoglobin concentration was measured in duplicate (HemoCue analyzer Hb201+, HemoCue, Sweden) and hemoglobin mass was calculated using regression equations with fat‐free mass, height, and sex (Oberholzer et al., 2024). Blood volume, plasma volume, and red cell volume were calculated using estimated hemoglobin mass, hematocrit, and hemoglobin concentration (Siebenmann et al., 2017). The average coefficient of variation for two repeat measurements 1 h apart in a subset of participants (*n* = 4) was 1.8% hematocrit, 0.3% for hemoglobin concentration, and 0.3% for blood volume.

### 2.6 Determination of body hypohydration

As our aim was to determine whether hypohydration affects perceptual responses to BFR exercise and not if 24 h of fluid restriction induces hypohydration per se, participants who did not accomplish hypohydration after the fluid restriction protocol were excluded. To account for variability and measurement error, a 1% body mass loss was used to address day‐to‐day fluctuations when hydrated (Cheuvront et al., 2004), and a 1.8% plasma volume loss accounted for errors in plasma volume estimation. Therefore, only participants who exhibited a ≥1% decrease in body mass and ≥1.8% decrease in plasma volume following fluid restriction were included in the analysis.

### 2.7 Statistical analyses

Data were analyzed using GraphPad Prism (Version 10.2.3, GraphPad Software, United States of America), with alpha set *a priori* at *p* = 0.05. A 2 (condition) × 5 (time) linear mixed model was performed for both RPE and leg pain during the BFR exercise sets. A 2 (condition) × 4 (time) linear mixed model was also performed for the rest sets. If a significant interaction was observed, post hoc tests were completed using the šídák correction for multiple comparisons. A three‐way ANOVA was performed to evaluate the interaction and exploratory relationship between biological sex, hydration, and BFR exercise. During the BFR exercise sets, a 2 (condition) × 2 (sex) × 3 (time) ANOVA was performed for both RPE and leg pain in only the “full” data set. Correlations between the change in RPE and leg pain and indices of hydration were performed using Spearman's nonparametric tests. Hematological values, urine specific gravity, and body mass were analyzed using a two‐tailed paired *t*‐tests. Sex‐based comparisons for physical characteristics and V̇O_2_ max were analyzed using unpaired *t*‐tests. Data are expressed as mean ± standard deviation.

## 3 RESULTS

Three participants were lost due to illness (*n* = 1), personal reasons (*n* = 1), and due to discomfort related to BFR exercise (*n* = 1) resulting in 31 participants who completed all the study visits.

Four participants (females *n* = 3, males *n* = 1) did not complete the protocol while hypohydrated because of syncopal symptoms. Symptoms were expressed in the second BFR rest period (*n* = 2), the fourth BFR exercise bout (*n* = 1), or the fourth BFR rest period (*n* = 1). These individuals completed the entire protocol without expressing symptoms during the hydrated visit. Of the individuals that experienced pre‐syncope symptoms, three out of four had their hypohydrated visit first.

Seventeen participants (males = 10, females = 7) were classified as hypohydrated after the 24 h fluid restriction (Figure S1 DOI: https://doi.org/10.6084/m9.figshare.27897456.v1). Participants included in this study were recreationally active with expected differences by sex in anthropometrics, hematological variables, and V̇O_2_ max relative to body mass, but not fat‐free mass (Table 1). Markers of body hydration for each visit can be found in Table 2. The LOP was similar between hydrated and hypohydrated visits.

**TABLE 1:** Participant characteristics from the introductory visit.

	Females (*n* = 7)	Males (*n* = 10)	Total (*n* = 17)
Age (years)	21 ± 2	24 ± 4	23 ± 3
Height (cm)	164 ± 6	177 ± 7*	171.7 ± 8.9
Body mass (kg)	61.3 ± 6.9	79.6 ± 11.2*	72.1 ± 13.2
Body fat (%)	27 ± 4	17 ± 5*	21 ± 7
Fat free mass (kg)	44.4 ± 3.7	65.7 ± 7.8*	56.9 ± 12.5
Body mass index (kg/m^2^)	22.7 ± 1.7	25.4 ± 2.9*	24.3 ± 2.8
Hemoglobin mass (g)	614 ± 48	954 ± 93*	814 ± 188
Peak watts			
Absolute (W)	220 ± 49	326 ± 72*	282 ± 82
Relative to body mass (W∙kg^−1^)	3.6 ± 0.8	4.1 ± 0.8	3.9 ± 0.8
Relative to fat‐free mass (W∙kg^−1^)	4.9 ± 1.0	5.0 ± 1.0	5.0 ± 1.0
V̇O_2_ max			
Absolute (L∙min^−1^)	2.6 ± 0.5	4.1 ± 0.8*	3.5 ± 1.1
Relative to body mass (mL∙kg^−1^∙min^−1^)	41.8 ± 7.6	52.0 ± 8.0*	47.8 ± 9.2
Relative to fat‐free mass (mL∙kg^−1^∙min^−1^)	57.6 ± 9.9	62.9 ± 10.6	60.7 ± 10.3

*Note*: Values are represented by mean ± standard deviation. An asterisk (*) is used to denote significance (*p* < 0.05) between males and females.

**TABLE 2:** Metrics of body hydration and limb occlusion pressure (LOP) during both hydrated and hypohydrated conditions.

	Hydrated	Dehydrated	*p*‐value	Percent change
Body mass (kg)	72.2 ± 13.0	70.6 ± 11.0	**<0.0001**	−2.3 ± 0.7
Urine‐specific gravity	1.01 ± 0.009	1.025 ± 0.002	**<0.0001**	+1.5 ± 0.9
Hemoglobin concentration (g/dL)	14.1 ± 1.3	14.8 ± 1.4	**<0.0001**	+4.4 ± 3.4
Hematocrit (%)	40.3 ± 4.0	42.2 ± 3.8	**<0.0001**	+4.7 ± 2.6
Blood volume (mL)	5712 ± 1017	5476 ± 978	**<0.0001**	−4.1 ± 3.1
Plasma volume (mL)	3387 ± 525	3148 ± 496	**<0.0001**	−7.0 ± 3.4
Red blood cell volume (mL)	2325 ± 570	2328 ± 551	0.9	+0.4 ± 3.8
LOP (mmHg)	210 ± 22	211 ± 27	0.8	+0.3 ± 7.4

*Note*: Values are represented by mean ± standard deviation (*n* = 17, males = 10 and females = 7). A negative percent change indicates a reduction during the hypohydrated visit and vice versa for a positive percent change.

**FIGURE 2:**
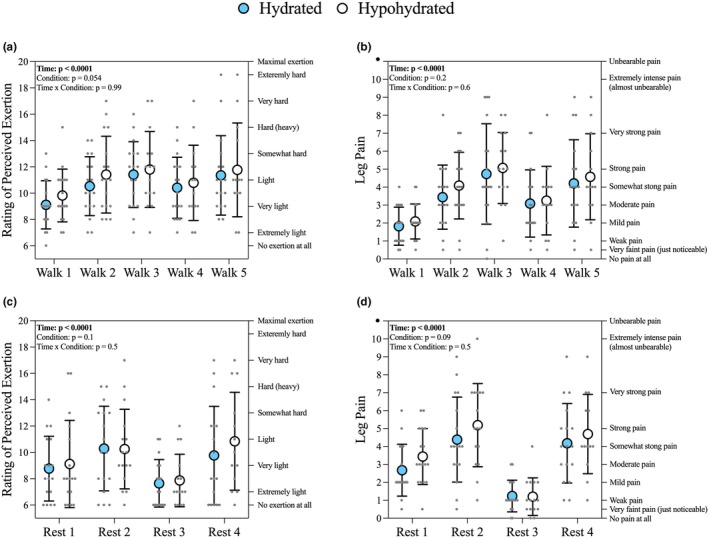
The influence of hypohydration on perceived exertion and leg pain during the BFR exercise intervention. (a) Rating of perceived exertion (RPE) during the BFR exercise sets. (b) Leg pain during the BFR exercise sets. (c) RPE during the rest periods. (d) Leg pain during the rest periods. Tourniquets were inflated throughout except for Rest 3. Blue and white circles represent mean hydrated and hypohydrated visit values, respectively and error bars represent the standard deviation. Individual responses are shown as gray circles (*n* = 13‐17/timepoint). Verbal anchors for RPE and leg pain are on the right y‐axis.

Hydration status did not change how participants rated leg pain and RPE across the BFR exercise set and rest periods (Figure 2). However, there was a tendency for the average RPE to be increased during exercise when hypohydrated (Hydrated: 10.6 ± 0.9, vs. Hypohydrated: 11.1 ± 0.8, *p* = 0.054, Figure 2a). Average leg pain was similar during exercise (Hydrated: 3.5 ± 1.1, vs. Hypohydrated: 3.8 ± 1.2, *p* = 0.2, Figure 2b). Hypohydration had a limited effect on average RPE and leg pain during the rest periods (Figure 2c,d).

**FIGURE 3:**
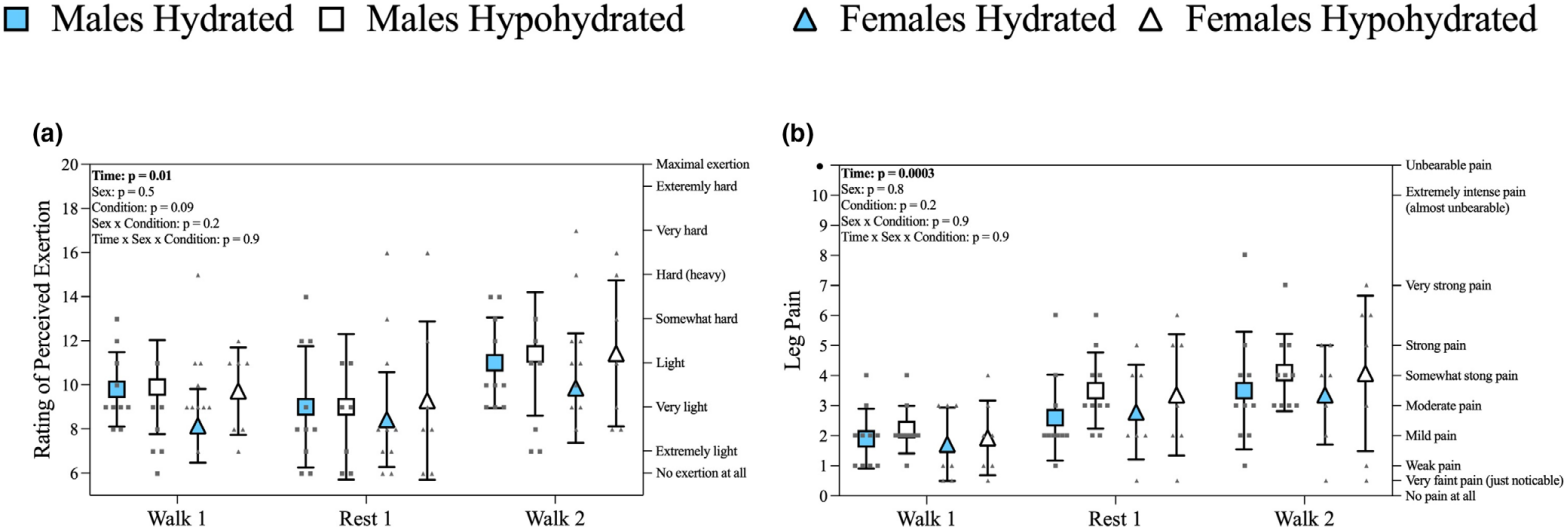
Exploratory analysis of the influence of hypohydration and sex on perceived exertion and leg pain during the BFR exercise intervention, prior to reported adverse responses of four participants leading to the inability to complete the entire exercise protocol. (a) rating of perceived exertion (RPE) during BFR exercise. (b) Leg pain during the BFR exercise. Males are represented as squares and females as triangles. The blue and white symbols represent the mean hydrated and hypohydrated visit values, respectively and error bars represent the standard deviation. Individual male responses are gray squares (*n* = 10) and individual female responses are gray triangles (*n* = 7). Verbal anchors for RPE and leg pain are on the right y‐axis.

There was no significant interaction between hypohydration and sex on perceptual responses during the BFR intervention (Figure 3). The change in metrics of hydration status did not correlate with the average changes in RPE or leg pain throughout the BFR intervention (Table 3). These relationships were not significant regardless of whether the analysis was separated by sex.

**TABLE 3:** Spearman's correlations metrics of hydration status and rating of perceived exertion or leg pain.

	Males (*n* = 10)	Females (*n* = 7)	Total (*n* = 17)
Mean change in rating of perceived exertion			
% change in body mass	ρ = −0.40, *p* = 0.3	ρ = −0.43, *p* = 0.4	ρ = −0.41, *p* = 0.1
% change in plasma volume	ρ = 0.26, *p* = 0.5	ρ = −0.39, *p* = 0.4	ρ = −0.05, *p* = 0.9
% change in blood volume	ρ = 0.28, *p* = 0.4	ρ = −0.46, *p* = 0.3	ρ = −0.07, *p* = 0.8
Change in urine specific gravity	ρ = 0.31, *p* = 0.4	ρ = −0.12, *p* = 0.8	ρ = 0.19, *p* = 0.5
Mean change in leg pain			
% change in body mass	ρ = −0.10, *p* = 0.8	ρ = −0.21, *p* = 0.7	ρ = −0.12, *p* = 0.7
% change in plasma volume	ρ = −0.02, *p* = 0.95	ρ = −0.21, *p* = 0.7	ρ = −0.13, *p* = 0.6
% change in blood volume	ρ = 0.20, *p* = 0.6	ρ = −0.43, *p* = 0.4	ρ = −0.14, *p* = 0.6
Change in urine specific gravity	ρ = −0.06, *p* = 0.9	ρ = 0.14, *p* = 0.8	ρ = 0.04, *p* = 0.9

*Note*: Values are represented by different metrics of hydration status versus either the mean change in rating of perceived exertion or the mean change in leg pain between the hydrated and hypohydrated visit. This data table takes an exploratory view on sex, as such it only influences the “full” data set prior to participant dropout. Mean change in RPE and pain is only calculated over walk 1, rest 1, and walk 2.

## 4 DISCUSSION

This study tested whether hydration status affected the perceived effort and leg pain responses during BFR exercise. The main novel finding was that hypohydration did not clearly affect RPE nor leg pain during the BFR exercise and rest periods. Our data also support that biological sex and common estimates of body hydration status were not related to the hypohydrated perceptual responses during the BFR intervention. However, four individuals did not complete the protocol due to pre‐syncopal symptoms—all during the hypohydrated trial—suggesting an individualized approach is needed to reduce undesirable outcomes.

### 4.1 Influence of hypohydration on perceptual responses during BFR exercise

Previous studies indicate that while RPE increases with prolonged exercise under progressive hypohydration, it remains unaffected during short‐duration activities, such as 2 min of handgrip exercise (Watso et al., 2019, 2020). Our findings align with this, suggesting that short‐duration exercise, with or without BFR, has minimal impact on perceived exertion while hypohydrated. This may be explained by the absence of significant core temperature increases, which are associated with elevated RPE during prolonged exercise (Nybo et al., 2002).

Our results differ from previous studies that report an increased pain sensitivity/intensity after 24 h fluid restriction during either post‐exercise (handgrip) circulatory occlusion or a cold pressure test (Bear et al., 2016; Tan et al., 2022) and in hypohydrated cyclists who completed an ultra‐endurance event (Moyen et al., 2015). These results may be, in part, attributed to methodological differences in pain quantifications. For example, previous studies report either minimal detectable or maximal tolerable pain sensations (Bear et al., 2016; Tan et al., 2022), whereas the current study collected pain ratings without the identification of a minimal detectable or “tolerable” pain sensation. Anecdotally, we believe if participants were instructed before the BFR exercise that the test would end when the pain exceeds a “tolerable” amount, individuals would have biased themselves to not completing the entire protocol due to the discomfort associated with this modality. As such, the discrepancies may be related to the relative pain intensities elicited between studies. For instance, we observed that BFR caused on average a “somewhat strong pain” to “very strong pain” within the first 7 min (regardless of hydration status), whereas ultra‐endurance cycling produced only “weak” to “mild pain” using the same pain rating scale (Moyen et al., 2015). This suggests a possible “ceiling effect”, where our protocol elicited greater pain sensations such that any hydration‐related differences become difficult to subjectively detect.

### 4.2 Influence of hypohydration and sex on perceptual responses during BFR exercise

Recent work supports that females experience a more rapid increase in RPE and pain during BFR exercise compared to males (McClean et al., 2023). Although not a primary aim of this study, our findings do not support that RPE and pain responses are influenced differently across sexes by hypohydration. However, four individuals—three of whom were females—did not complete the entire exercise protocol hypohydrated due to pre‐syncopal symptoms. In line with our findings, a proportion of practitioners have reported syncopal symptoms as a side effect when using BFR exercise (Colapietro et al., 2024; Patterson & Brandner, 2018). Interestingly, there was no evidence of greater hypohydration with these four individuals compared to the 13 individuals who did not experience pre‐syncope symptoms (e.g., percent change in body mass: −2.6 ± 0.5% vs. −2.3 ± 0.8%, plasma volume: −7.1 ± 3.2% vs. −7.0 ± 3.6%, and blood volume: ‐3.4 ± 3.5% vs. −4.3 ± 3.1%). Despite limited evidence in our data to explain why these four individuals only experienced syncopal symptoms while hypohydrated, previous research suggests a distinct subset of individuals have a hypotensive response to the stimulation of type III/IV afferent nerves (Fazalbhoy et al., 2012). Collectively, hydration status may alter the interstitial metabolites that stimulate type III/IV afferent nerve activity during BFR exercise and increase the susceptibility of a subset of individuals to adverse responses. Future work could seek to explore whether individuals who have a hypotensive response to experimentally induced pain also have a greater risk of adverse responses like pre‐syncope symptoms during BFR exercise.

### 4.3 Study considerations

This study has a few considerations that should be considered when interpreting results. First, we introduced only mild hypohydration (~2% loss in body mass), which is considered the “cut‐off” for exercise performance impairments (Cheuvront et al., 2010) and may not capture more consistent effects as seen with more severe hypohydration (Montain & Coyle, 1992). Nonetheless, these findings are still ecologically valid as individuals could be inadvertently performing BFR exercise while mildly hypohydrated. Second, our findings of no sex differences should be interpreted with caution due to the relatively low sample size; however, our preliminary results suggest that any differences would be small. Lastly, due to our study design, individuals were not blinded to the expected hydration state. However, given the overall findings, the influence of bias appears low.

## 5 CONCLUSION

In conclusion, mild hypohydration did not clearly alter the perception of exertion and leg pain during BFR exercise. However, given the potential for BFR exercise as a training and rehabilitative modality, it should still be advised for individuals to arrive hydrated to reduce potential undesirable outcomes, including pre‐syncopal symptoms.

## AUTHOR CONTRIBUTIONS

JCM, CP, GCL, MMG, KT, REJP, EMBC, and AAR contributed to the collection of data. JCM, CP, and GCL analyzed the data. JCM prepared the figures. JCM drafted the manuscript. CP and JFB designed the study. All authors interpreted the results and approved the final version of the manuscript.

## FUNDING INFORMATION

No sources of funding were used for this study.

## CONFLICT OF INTEREST STATEMENT

The authors declare no conflicts of interest.

## ETHICS STATEMENT

This study was approved by the University of Guelph research ethics board.

## Supporting information


Figure S1.

## References

[phy270343-bib-0001] Armstrong, L. E. , Costill, D. L. , & Fink, W. J. (1985). Influence of diuretic‐induced dehydration on competitive running performance. Medicine and Science in Sports and Exercise, 17, 456–461. 10.1249/00005768-198508000-00009 4033401

[phy270343-bib-0002] Bear, T. , Philipp, M. , Hill, S. , & Mündel, T. (2016). A preliminary study on how hypohydration affects pain perception. Psychophysiology, 53, 605–610. 10.1111/psyp.12610 26785699

[phy270343-bib-0003] Bergevin, M. , Steele, J. , Payen De La Garanderie, M. , Feral‐Basin, C. , Marcora, S. M. , Rainville, P. , Caron, J. G. , & Pageaux, B. (2023). Pharmacological blockade of muscle afferents and perception of effort: A systematic review with meta‐analysis. Sports Medicine, 53, 415–435. 10.1007/s40279-022-01762-4 36318384

[phy270343-bib-0004] Borg, G. A. (1982). Psychophysical bases of perceived exertion. Medicine & Science in Sports & Exercise, 14, 377–381. 10.1249/00005768-198205000-00012 7154893

[phy270343-bib-0005] Cerqueira, M. S. , Costa, E. C. , Santos Oliveira, R. , Pereira, R. , & Brito Vieira, W. H. (2021). Blood flow restriction training: To adjust or not adjust the cuff pressure over an intervention period? Frontiers in Physiology, 12, 678407. 10.3389/fphys.2021.678407 34262476 PMC8273389

[phy270343-bib-0006] Chaplin, H. , & Mollison, P. L. (1952). Correction for plasma trapped in the red cell column of the hematocrit. Blood, 7, 1227–1238. 10.1182/blood.v7.12.1227.1227 12997539

[phy270343-bib-0007] Chaplin, H. , Mollison, P. L. , & Vetter, H. (1953). The body/venous hematocrit ratio: Its constancy over a wide hematocrit range. The Journal of Clinical Investigation, 32, 1309–1316. 10.1172/JCI102859 13108998 PMC438476

[phy270343-bib-0008] Cheuvront, S. N. , Carter, R. , Montain, S. J. , & Sawka, M. N. (2004). Daily body mass variability and stability in active men undergoing exercise‐heat stress. International Journal of Sport Nutrition and Exercise Metabolism, 14, 532–540. 10.1123/ijsnem.14.5.532 15673099

[phy270343-bib-0009] Cheuvront, S. N. , Kenefick, R. W. , Montain, S. J. , & Sawka, M. N. (2010). Mechanisms of aerobic performance impairment with heat stress and dehydration. Journal of Applied Physiology, 109, 1989–1995. 10.1152/japplphysiol.00367.2010 20689090

[phy270343-bib-0010] Cognetti, D. J. , Sheean, A. J. , & Owens, J. G. (2022). Blood flow restriction therapy and its use for rehabilitation and return to sport: Physiology, application, and guidelines for implementation. Arthroscopy, Sports Medicine, and Rehabilitation, 4, e71–e76. 10.1016/j.asmr.2021.09.025 35141538 PMC8811521

[phy270343-bib-0011] Colapietro, M. A. , Lee, J. Z. , & Vairo, G. L. (2024). Survey of blood flow restriction training applications in sports medicine and performance practice across North America. Journal of Strength and Conditioning Research, 38, 856–863. 10.1519/JSC.0000000000004702 38241466

[phy270343-bib-0012] Cook, D. B. , O'Connor, P. J. , Oliver, S. E. , & Lee, Y. (1998). Sex differences in naturally occurring leg muscle pain and exertion during maximal cycle ergometry. The International Journal of Neuroscience, 95, 183–202. 10.3109/00207459809003340 9777439

[phy270343-bib-0013] Fazalbhoy, A. , Birznieks, I. , & Macefield, V. G. (2012). Individual differences in the cardiovascular responses to tonic muscle pain: Parallel increases or decreases in muscle sympathetic nerve activity, blood pressure and heart rate. Experimental Physiology, 97, 1084–1092. 10.1113/expphysiol.2012.066191 22581744

[phy270343-bib-0014] Fisher, A. C. , Domm, M. A. , & Wuest, D. A. (1988). Adherence to sports‐injury rehabilitation programs. The Physician and Sportsmedicine, 16, 47–52. 10.1080/00913847.1988.11709551 27403824

[phy270343-bib-0015] Fitschen, P. J. , Kistler, B. M. , Jeong, J. H. , Chung, H. R. , Wu, P. T. , Walsh, M. J. , & Wilund, K. R. (2014). Perceptual effects and efficacy of intermittent or continuous blood flow restriction resistance training. Clinical Physiology and Functional Imaging, 34, 356–363. 10.1111/cpf.12100 24666729

[phy270343-bib-0016] Jack, K. , McLean, S. M. , Moffett, J. K. , & Gardiner, E. (2010). Barriers to treatment adherence in physiotherapy outpatient clinics: A systematic review. Manual Therapy, 15, 220–228. 10.1016/j.math.2009.12.004 20163979 PMC2923776

[phy270343-bib-0017] Logan‐Sprenger, H. M. , Heigenhauser, G. J. F. , Jones, G. L. , & Spriet, L. L. (2015). The effect of dehydration on muscle metabolism and time trial performance during prolonged cycling in males. Physiological Reports, 3, e12483. 10.14814/phy2.12483 26296770 PMC4562569

[phy270343-bib-0018] Masri, B. A. , Day, B. , Younger, A. S. E. , & Jeyasurya, J. (2016). Technique for measuring limb occlusion pressure that facilitates personalized tourniquet systems: A randomized trial. Journal of Medical and Biological Engineering, 36, 644–650. 10.1007/s40846-016-0173-5 27853415 PMC5083760

[phy270343-bib-0019] McClean, Z. J. , Young, A. , Pohl, A. J. , Fine, N. M. , Burr, J. F. , MacInnis, M. , & Aboodarda, S. J. (2023). Blood flow restriction during high‐intensity interval cycling exacerbates psychophysiological responses to a greater extent in females than males. Journal of Applied Physiology, 134, 596–609. 10.1152/japplphysiol.00567.2022 36701480

[phy270343-bib-0020] Montain, S. J. , & Coyle, E. F. (1992). Influence of graded dehydration on hyperthermia and cardiovascular drift during exercise. Journal of Applied Physiology, 73, 1340–1350. 10.1152/jappl.1992.73.4.1340 1447078

[phy270343-bib-0021] Moyen, N. E. , Ganio, M. S. , Wiersma, L. D. , Kavouras, S. A. , Gray, M. , McDermott, B. P. , Adams, J. D. , Binns, A. P. , Judelson, D. A. , McKenzie, A. L. , Johnson, E. C. , Muñoz, C. X. , Kunces, L. J. , & Armstrong, L. E. (2015). Hydration status affects mood state and pain sensation during ultra‐endurance cycling. Journal of Sports Sciences, 33, 1962–1969. 10.1080/02640414.2015.1021275 25793570

[phy270343-bib-0022] Nybo, L. , Møller, K. , Volianitis, S. , Nielsen, B. , & Secher, N. H. (2002). Effects of hyperthermia on cerebral blood flow and metabolism during prolonged exercise in humans. Journal of Applied Physiology, 93, 58–64. 10.1152/japplphysiol.00049.2002 12070186

[phy270343-bib-0023] Oberholzer, L. , Montero, D. , Robach, P. , Siebenmann, C. , Ryrsøe, C. K. , Bonne, T. C. , Breenfeldt Andersen, A. , Bejder, J. , Karlsen, T. , Edvardsen, E. , Rønnestad, B. R. , Hamarsland, H. , Cepeda‐Lopez, A. C. , Rittweger, J. , Treff, G. , Ahlgrim, C. , Almquist, N. W. , Hallén, J. , & Lundby, C. (2024). Determinants and reference values for blood volume and total hemoglobin mass in women and men. American Journal of Hematology, 99, 88–98. 10.1002/ajh.27162 38032792

[phy270343-bib-0024] Patterson, S. D. , & Brandner, C. R. (2018). The role of blood flow restriction training for applied practitioners: A questionnaire‐based survey. Journal of Sports Sciences, 36, 123–130. 10.1080/02640414.2017.1284341 28143359

[phy270343-bib-0025] Patterson, S. D. , Hughes, L. , Warmington, S. , Burr, J. , Scott, B. R. , Owens, J. , Abe, T. , Nielsen, J. L. , Libardi, C. A. , Laurentino, G. , Neto, G. R. , Brandner, C. , Martin‐Hernandez, J. , & Loenneke, J. (2019). Blood flow restriction exercise: Considerations of methodology, application, and safety. Frontiers in Physiology, 10, 533. 10.3389/fphys.2019.00533 31156448 PMC6530612

[phy270343-bib-0027] Pollak, K. A. , Swenson, J. D. , Vanhaitsma, T. A. , Hughen, R. W. , Jo, D. , White, A. T. , Light, K. C. , Schweinhardt, P. , Amann, M. , & Light, A. R. (2014). Exogenously applied muscle metabolites synergistically evoke sensations of muscle fatigue and pain in human subjects. Experimental Physiology, 99, 368–380. 10.1113/expphysiol.2013.075812 24142455 PMC3946674

[phy270343-bib-0028] Rossow, L. M. , Fahs, C. A. , Loenneke, J. P. , Thiebaud, R. S. , Sherk, V. D. , Abe, T. , & Bemben, M. G. (2012). Cardiovascular and perceptual responses to blood‐flow‐restricted resistance exercise with differing restrictive cuffs. Clinical Physiology and Functional Imaging, 32, 331–337. 10.1111/j.1475-097X.2012.01131.x 22856338

[phy270343-bib-0029] Shirreffs, S. M. , Merson, S. J. , Fraser, S. M. , & Archer, D. T. (2004). The effects of fluid restriction on hydration status and subjective feelings in man. The British Journal of Nutrition, 91, 951–958. 10.1079/BJN20041149 15182398

[phy270343-bib-0030] Siebenmann, C. , Keiser, S. , Robach, P. , & Lundby, C. (2017). CORP: The assessment of total hemoglobin mass by carbon monoxide rebreathing. Journal of Applied Physiology, 123, 645–654. 10.1152/japplphysiol.00185.2017 28663373

[phy270343-bib-0031] Tan, B. , Philipp, M. C. , Che Muhamed, A. M. , & Mündel, T. (2022). Hypohydration but not menstrual phase influences pain perception in healthy women. Journal of Applied Physiology, 132, 611–621. 10.1152/japplphysiol.00402.2021 35085028

[phy270343-bib-0032] Tankersley, C. G. , Zappe, D. H. , Meister, T. G. , & Kenney, W. L. (1985). Hypohydration affects forearm vascular conductance independent of heart rate during exercise. Journal of Applied Physiology (Bethesda, Md.), 73, 1232–1237. 10.1152/jappl.1992.73.4.1232 1447064

[phy270343-bib-0033] Thompson, K. M. A. , Gamble, A. S. D. , Kontro, H. , Lee, J. B. , & Burr, J. F. (2024). Low‐ and high‐volume blood‐flow restriction treadmill walking both improve maximal aerobic capacity independently of blood volume. Scandinavian Journal of Medicine & Science in Sports, 34, e14534. 10.1111/sms.14534 37961932

[phy270343-bib-0034] Watso, J. C. , Babcock, M. C. , Robinson, A. T. , Migdal, K. U. , Wenner, M. M. , Stocker, S. D. , & Farquhar, W. B. (2019). Water deprivation does not augment sympathetic or pressor responses to sciatic afferent nerve stimulation in rats or to static exercise in humans. Journal of Applied Physiology, 127, 235–245. 10.1152/japplphysiol.00005.2019 31070954 PMC6692747

[phy270343-bib-0035] Watso, J. C. , Robinson, A. T. , Babcock, M. C. , Migdal, K. U. , Witman, M. A. H. , Wenner, M. M. , Stocker, S. D. , & Farquhar, W. B. (2020). Short‐term water deprivation attenuates the exercise pressor reflex in older female adults. Physiological Reports, 8, e14581. 10.14814/phy2.14581 32965797 PMC7510566

[phy270343-bib-0036] Weatherholt, A. , Beekley, M. , Greer, S. , Urtel, M. , & Mikesky, A. (2013). Modified Kaatsu training: Adaptations and subject perceptions. Medicine & Science in Sports & Exercise, 45, 952. 10.1249/MSS.0b013e31827ddb1f 23247712

