# Supplementary material for: The effects of body hydration on perceptual responses during blood flow restriction exercise
Source: Physiol Rep. 2025 Apr 22;13(8):e70343. doi: 10.14814/phy2.70343 (PMC12012723; doi:10.14814/phy2.70343)
Supplement: Supplementary file 1 — Figure S1. [file PHY2-13-e70343-s001.docx]

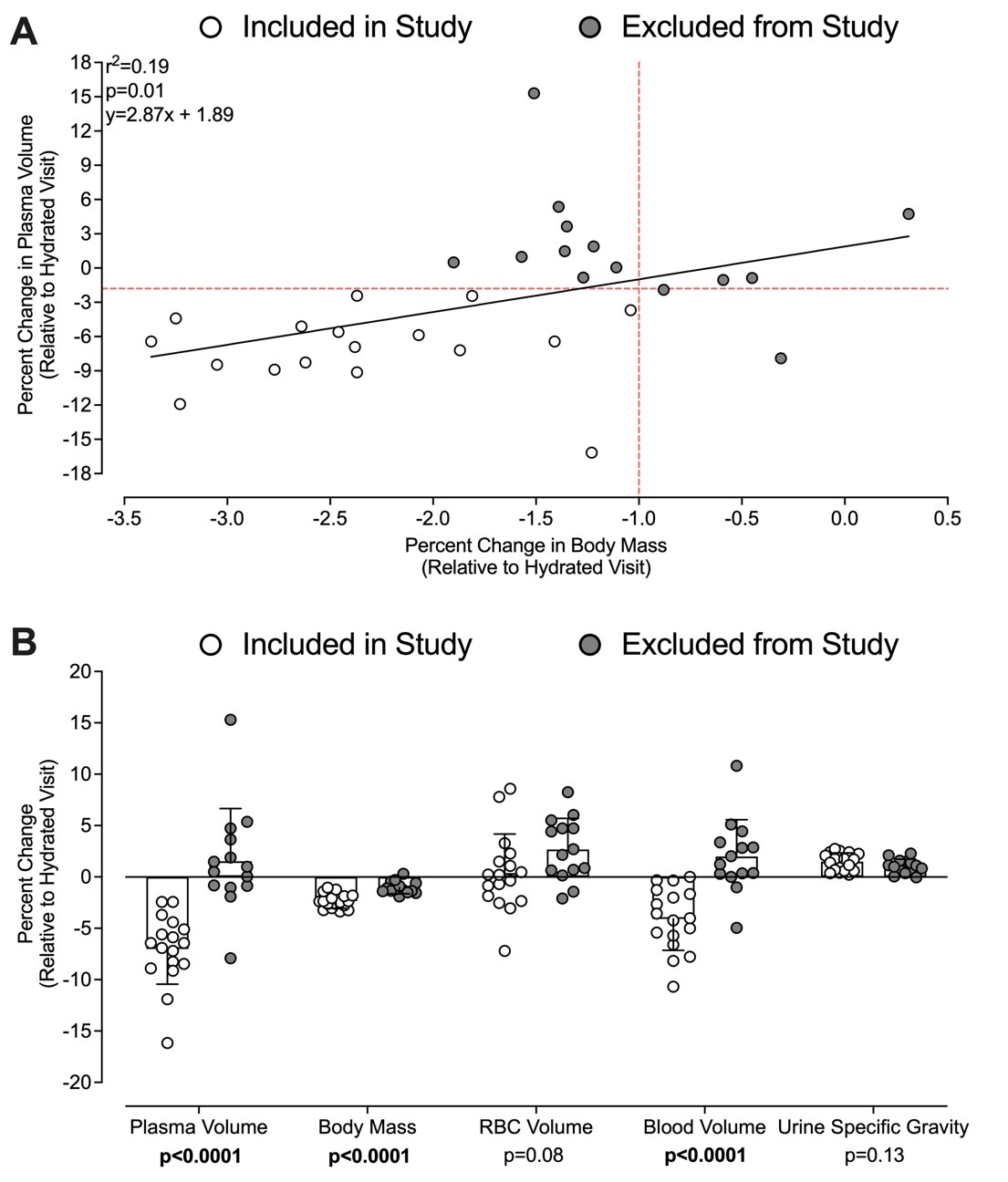


Supplementary Figure 1. (A) Percent change in body mass versus plasma volume of all participants (n=31) after 24 hours of fluid restriction. The solid black line represents the line of best fit with the r^2^, p-value, and equation of the line in the top left of the graph. This relationship is similar to controlled dehydration via sweating (y=2.8x+1.35)^1^. The red dashed lines represent -1% body mass (vertical line) and -1.8% plasma volume (horizontal line), which were used as criteria to classify participants as dehydrated. The individual white circles represent the participants included in the study who met both criteria (n=17) and the individual gray circles represent the participants excluded in the study who failed to meet both criteria (n=14). A -1% body mass loss was used to account for day-to-day variability in body mass when hydrated^2^. A -1.8% loss in plasma volume was used to reflect measurement error of estimating plasma volume. (B) Comparison of dehydration metrics between participants included and excluded with unpaired t-test values shown below each variable. Collectively, these data support those excluded from the study likely failed to dehydrate themselves following 24 hours of fluid restriction.

**References**:

1. Cheuvront, S. N., Kenefick, R. W., Montain, S. J. & Sawka, M. N. Mechanisms of aerobic performance impairment with heat stress and dehydration. *J. Appl. Physiol.* **109**, 1989–1995 (2010).

2. Cheuvront, S. N., Carter, R., Montain, S. J. & Sawka, M. N. Daily Body Mass Variability and Stability in Active Men Undergoing Exercise-Heat Stress. *Int. J. Sport Nutr. Exerc. Metab.* **14**, 532–540 (2004).
